# Supplementary material for: RefCell: multi-dimensional analysis of image-based high-throughput screens based on ‘typical cells’
Source: BMC Bioinformatics. 2018 Nov 16;19:427. doi: 10.1186/s12859-018-2454-1 (PMC6240236; doi:10.1186/s12859-018-2454-1)

# Additional file 1

## S1 Typical healthy and progeria cell nuclei

**
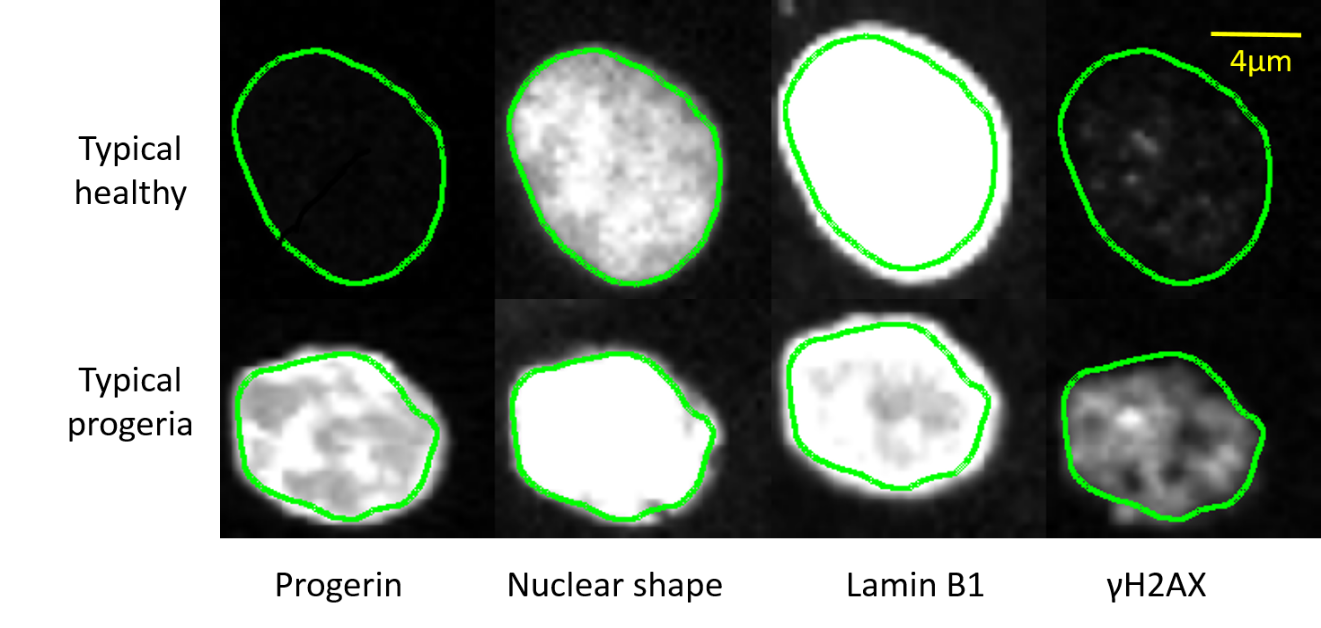
**

**Figure S1. Representative images of typical healthy (top row) and progeria (bottom row) nuclei.** Images are shown in four different channels: progerin, DAPI, lamin B1, and γH2AX. The outline of each nucleus (shown in green) was first extracted from the DAPI channel (nuclear shape) and then mapped onto the other three channels. As the images show, typical progeria nuclei have pronounced progerin expression, blebbed nuclear outlines, decreased lamin B1 expression, and high levels of DNA damage (γH2AX).

## S2 Sorting out the mismatch of images across different channels

In Fig 1 (a), Fig 6 and Fig S1, we noticed that nucleus in Lamin B1 channel tends to be larger than its counterpart in DAPI channel. In addition, there is also a shift in the image when we tried to overlay the outline segmented from DAPI channel directly onto Lamin B1 channel. Since our intensity measurements (measurements for Lamin B1, Progerin and γH2AX channel) are based on the outlines segmented in DAPI channel (we did this because cells used in our experiment are perturbed in lamin B1, progerin and γH2AX expression, causing them not reliable for outline segmentation), these two problems can lead to serious mistake. In order to sort out these two issues, we compared measurements between their values calculated based on outline segmented from DAPI channel and outline segmented in lamin B1 channel. A random sample of nuclei in GFP-progerin repressed control are used for this comparison, and four measurements (square root of area, boundary point intensity, mean intensity and standard deviation of intensity) are compared. Plotted in Fig S2 are the results. Each dot in Fig S2 represents a nucleus. As shown in Fig S2 (a), the size of nucleus measured in Lamin B1 channel is constantly larger than DAPI channel, with the average nuclear radius calculated in Lamin B1 channel ~ 600 nm longer than in DAPI channel. This difference may be due to the fact that DAPI attaches to DNA while Lamin B1 stain directly attaches to lamina that supports nuclear membrane. As to the shifting, since different cameras are used to capture images in different channels, even though the alignment of cameras was auto-corrected, there can still be slight shifts of direction. As shown in Fig S2 (b) – (c), the three intensity measurements are highly correlated between measurements calculated based on DAPI outline or Lamin B1 outline with correlation coefficients almost 1 (p value close to 0) for mean intensity and boundary point intensity. These results suggest the shift in direction is small and intensity measurement based on DAPI outline is a reliable replacement of direct measurement based on outlines segmented in lamin B1 channel. Since Lamin B1 (as well as progerin and γH2AX) is perturbed as its expression level decreases when progerin exists leading some nuclei invisible in lamin B1 channel, we performed lamin B1 (as well as progerin and γH2AX) measurements based on DAPI outline.


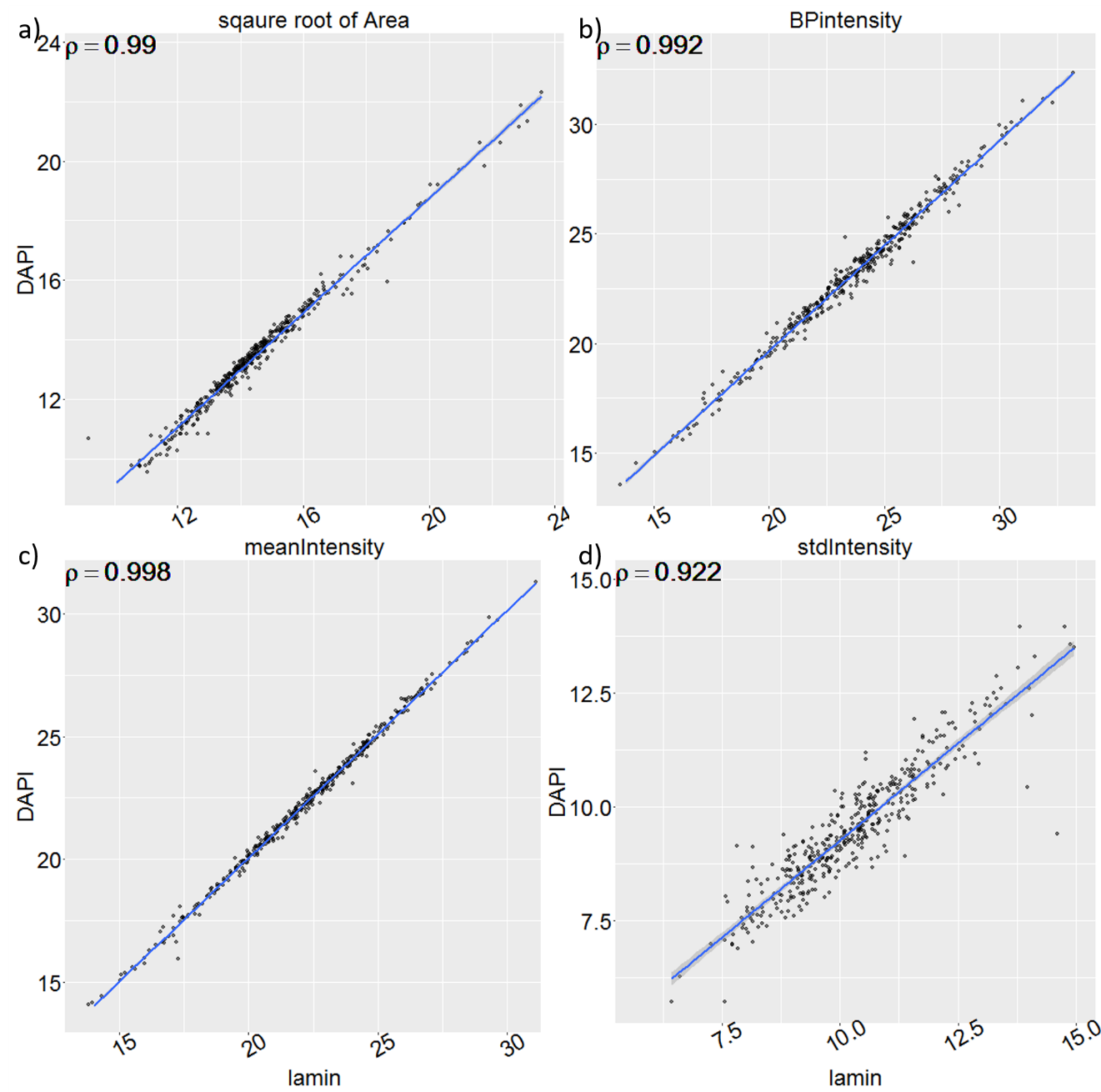


**Figure S2. Comparison between measurements obtained from outlines segmented in DAPI vs Lamin B1 channels.** Corresponding correlation coefficients are shown in the top left corner of each panel. BPintensity stands for boundary point intensity, which measures mean fluorescent intensity along nuclear boundary; meanIntensity stands for mean intensity inside nucleus; and stdIntensity is standard deviation of fluorescent intensity inside nucleus boundary.

## S3 Image processing and feature selection

The raw data for image-based HTS consists of fluorescence microscopy images. We studied cell nuclei imaged in four channels (see Fig S1). From the DAPI channel, we first analyzed nuclear shapes extracted with an active contour algorithm [38], from which we determined 12 shape metrics. Out of these, 5 measurements are *global metrics*, i.e. parameters that describe the overall shape of the boundary (area, perimeter, eccentricity, major and minor axis length), while the remaining 7 measurements are *local metrics*, sensitive to the local features of the shape (number of invaginations, standard deviation of the curvature, mean curvature, solidity, mean negative curvature, circularity, and tortuosity). For analysis, an online open source package (http://downloads.openmicroscopy.org/bio-formats/5.1.2/) was incorporated into the nuclear shape extraction algorithm [38] to read flex format images. Furthermore, to detect and remove overlapping nuclei from multiple cells, we set up outlier detection thresholds for area and solidity at two standard deviations from the mean, and discarded segmented nuclear outlines with area or solidity beyond the thresholds (about 6% of nuclei were discarded at this stage). The nuclear outlines extracted in the DAPI channel were mapped to the other 3 channels for analysis of fluorescence intensity, as shown by the green boundaries in Fig. S1. In each of the 3 other channels, we determined 3 metrics as basic characteristics of the intensity distribution in each nucleus: mean intensity, standard deviation of intensity, and mean intensity along the boundary. Therefore, in total, we obtained 21 metrics for each nucleus, of which 12 represent shape features and 9 represent 3 metrics for each of the 3 channels label lamin B1, progerin, and DNA damage (γH2AX).

As a starting point, to select meaningful metrics that differ between GFP-progerin expressing and repressed controls, we analyzed each metric separately using F-scores [40], defined as

$F(i)\equiv\frac{\left( {\bar{x_{i}}}^{(+)}-\bar{x_{i}} \right)^{2}+\left( {\bar{x_{i}}}^{(-)}-\bar{x_{i}} \right)^{2}}{\frac{1}{n_{+}-1}\sum_{k=1}^{n_{+}} \left( x_{k,i}^{(+)}-{\bar{x_{i}}}^{(+)} \right)^{2}+\frac{1}{n_{-}-1}\sum_{k=1}^{n_{-}} \left( x_{k,i}^{(-)}-{\bar{x_{i}}}^{(-)} \right)^{2}}$ $F(i)\equiv\frac{\left( {\bar{x_{i}}}^{(+)}-\bar{x_{i}} \right)^{2}+\left( {\bar{x_{i}}}^{(-)}-\bar{x_{i}} \right)^{2}}{\frac{1}{n_{+}-1}\sum_{k=1}^{n_{+}} \left( x_{k,i}^{(+)}-{\bar{x_{i}}}^{(+)} \right)^{2}+\frac{1}{n_{-}-1}\sum_{k=1}^{n_{-}} \left( x_{k,i}^{(-)}-{\bar{x_{i}}}^{(-)} \right)^{2}}$ (1)

where $x_{i}^{(+/-)}$ ${\bar{x_{i}}}^{(+/-)}$ is the mean of the *i*th measurement for GFP-progerin expressing and repressed controls, respectively; $\bar{x_{i}}$ is the mean of the *i*th measurement for both controls combined, and $n_{+/-}$ is the number of cells in GPF-progerin expressing and repressed controls. Using this procedure, we removed 6 shape metrics (eccentricity, minor axis length, major axis length, mean curvature, area, and perimeter), which yielded very low F-scores (<0.003), from further analysis. The F-scores for the included shape metrics ranged from 0.5 to 17.

## S4 Determination of parameters used in the selection of typical control cells

Typical cells for control cell type A are defined as cells that are closest to the center of the multi-dimensional distribution of all cells in type A. Hence, there are two parameters to be determined before typical cell selection: the definition of center, and the number of typical cells to be selected. In this paper, we tested 3 definitions of center: the mean, the median, and the global peak of each distribution. The peak of distribution was calculated using .find_peaks() function in R package openCyto [42]. We also tested 8 different numbers of typical cells: 100, 200, 300, 500, 1000, 2000, 3000, and 5000. 1500 cells that are next closest to the center (compare to typical cells) were selected as cross validation set (CV). The distance between each cell and the center of distribution was calculated using L1 Manhattan distance. Typical cells were selected independently in each channel, thus different cells may be selected as typical cells in different channels. We applied support vector machine (SVM) to classify typical healthy and typical progeria cells selected using all cells numbers and corresponding to all 3 definitions of center (in total 3X8=24 different conditions) for each replicate plate. After the classification, predictions were made for CV cells. The accuracy of training set (dashed lines) and cross validation set (solid line) for each condition in replicate plate 1 were plotted in Fig S3. We concluded from the results of all replicate plates that using mean as center definition and choosing 300 typical cells gave the best accuracy for our data.


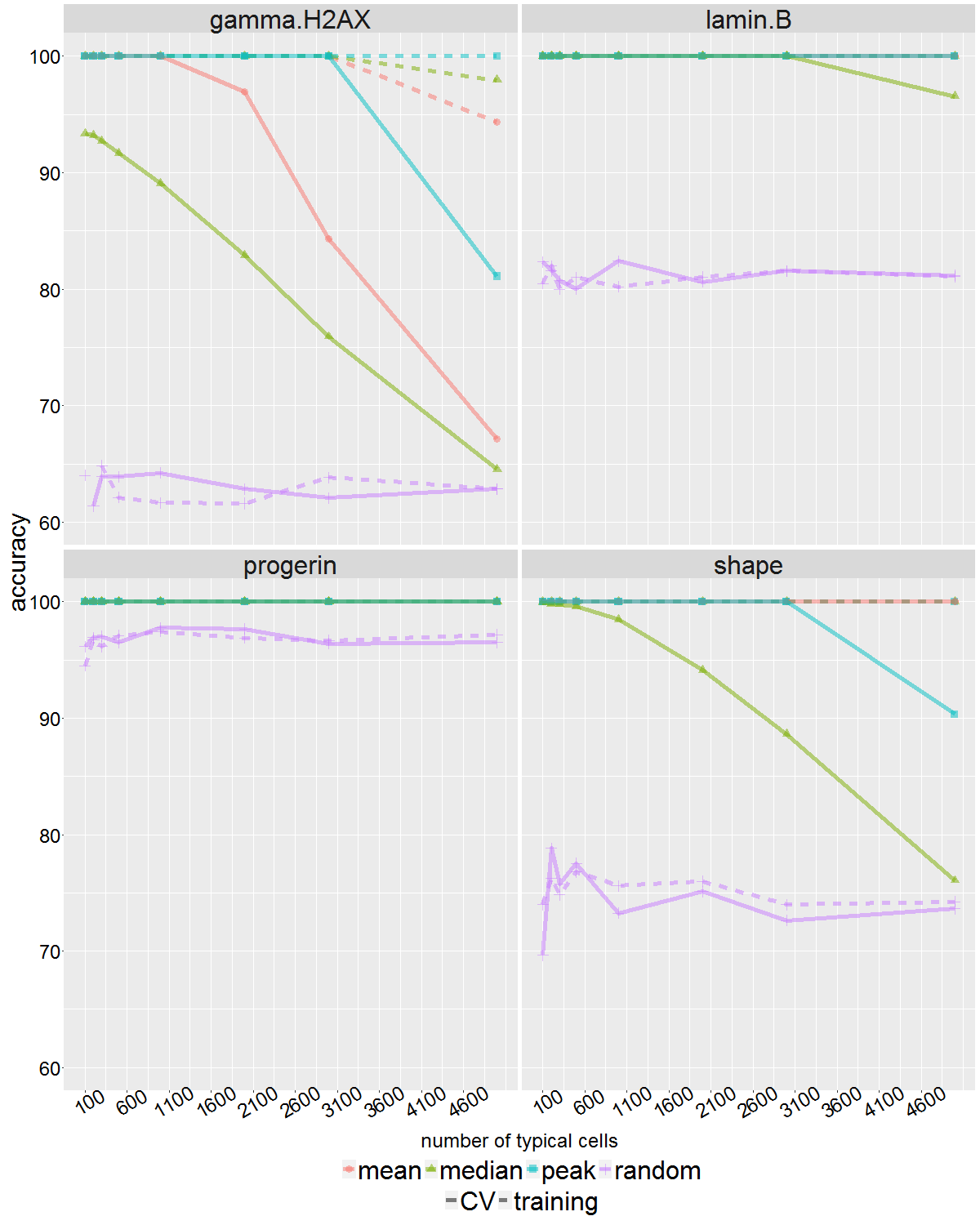


**Figure S3. Comparison of different center definition: mean (pink), median (green), peak (blue); and different typical cell numbers in plate 1.** For reference we also included randomly selected cells (purple). Vertical axis shows accuracy of classification, and horizontal axis is the selected typical cell number. Each subfigure plots results in one channel. Dashed line shows accuracy for training set, and solid line shows cross validation set. Notice that randomly selected cells consistently behave worst, and for all channels, mean and peak behaved equally well in this replicate, but overall mean behaves the best.

# S5 Relative weights of each measurement in the 4 channels


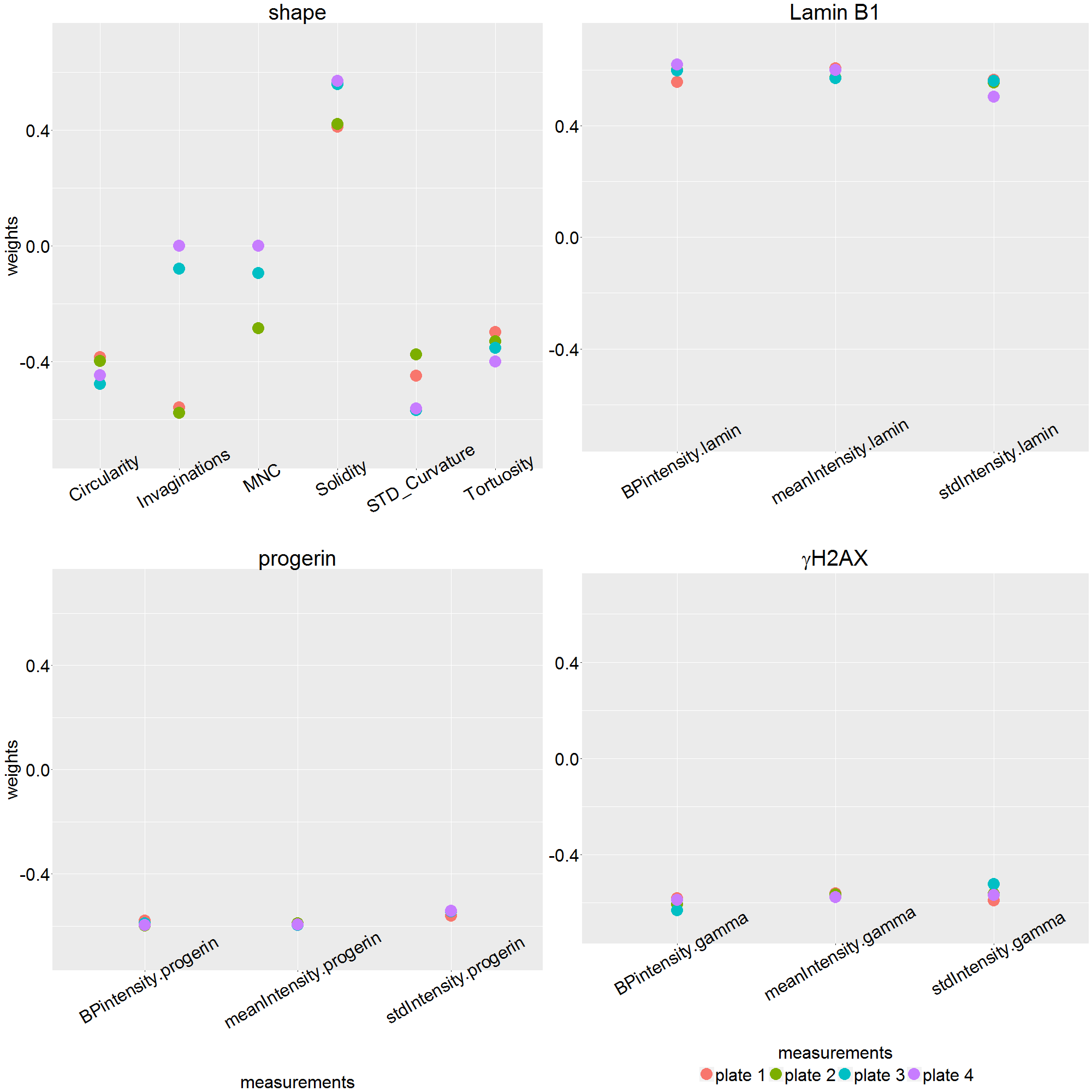


**Figure S4. Weights of each measurement in all channels, plates are labeled with different colors.** Absolute value of weights indicates the importance of the corresponding measurement in classifying typical healthy vs. typical progeria cells. Sign of the weights indicates in which control type the very measurement is higher. Negative sign means it’s higher in progeria controls, and vice versa. For example, solidity in shape channel has a positive sign, which means its value is higher in healthy control cells. And all measurements in progerin channel have a negative sign, this is to say all the 3 measurements have higher value in progeria controls, which is what we expected. Solidity is the most important measurement in shape channel. Weights for intensity measurements are quite similar with only small differences, and fluctuates over different plates.

## S6 Calculation of healthy-like cell percentage in each channel


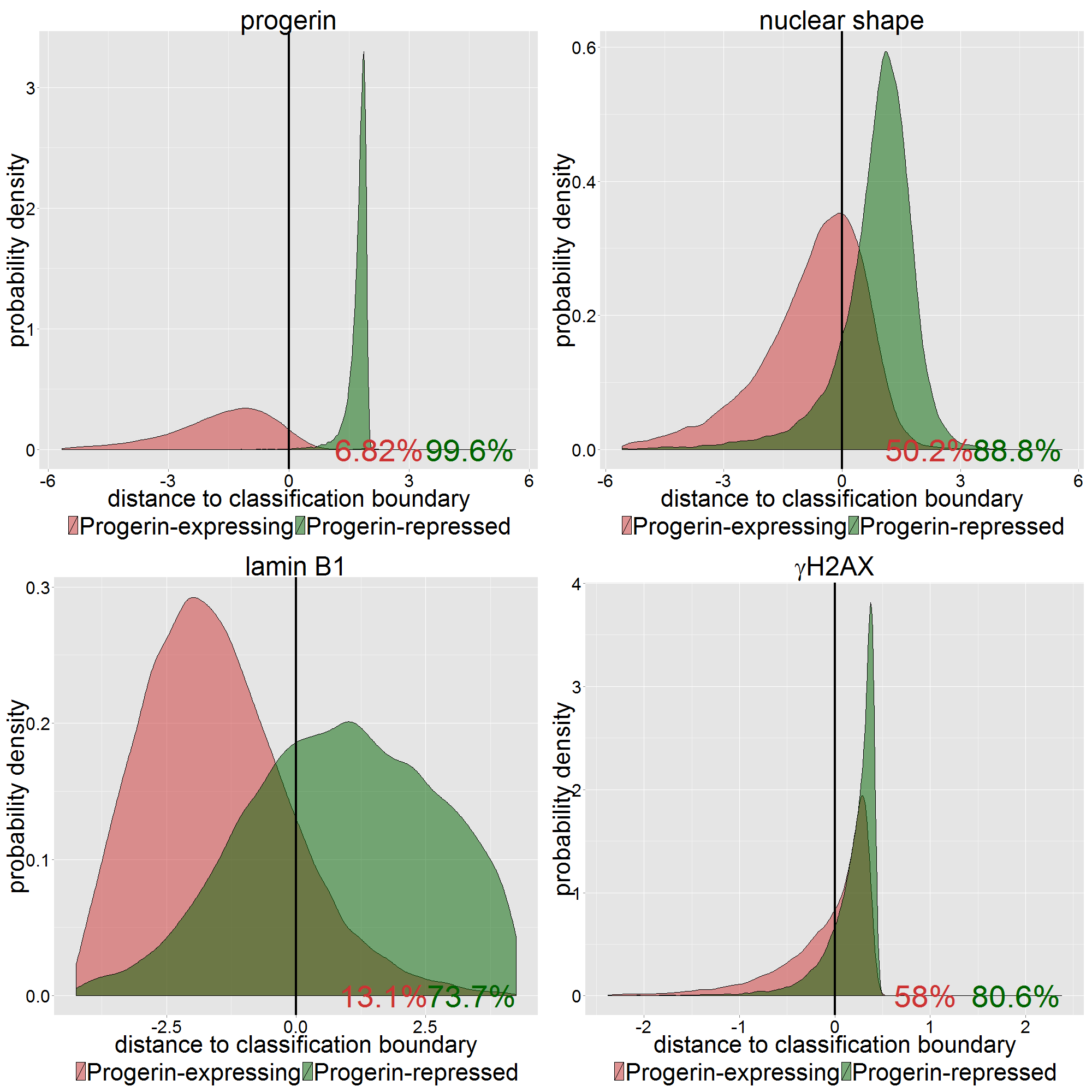


**Figure S5. Probability density distribution of all cells in GFP-progerin repressed (green) and GFP-progerin expressing (red) controls to the classification boundary in replicate plate 1.** In each panel, the vertical line indicates the classification boundary located at x=0. We show here the combination of cells in 12 GFP-progerin repressed control samples (green) and 12 GFP-progerin expressing control samples (red). As expected, the progerin channel is the most distinctive between GFP-progerin repressed and expressing controls. On the bottom right of each panel, we show the average percentage of healthy-like (green) and progeria-like (red) cells in each channel, calculated from the 4 replicate plates.


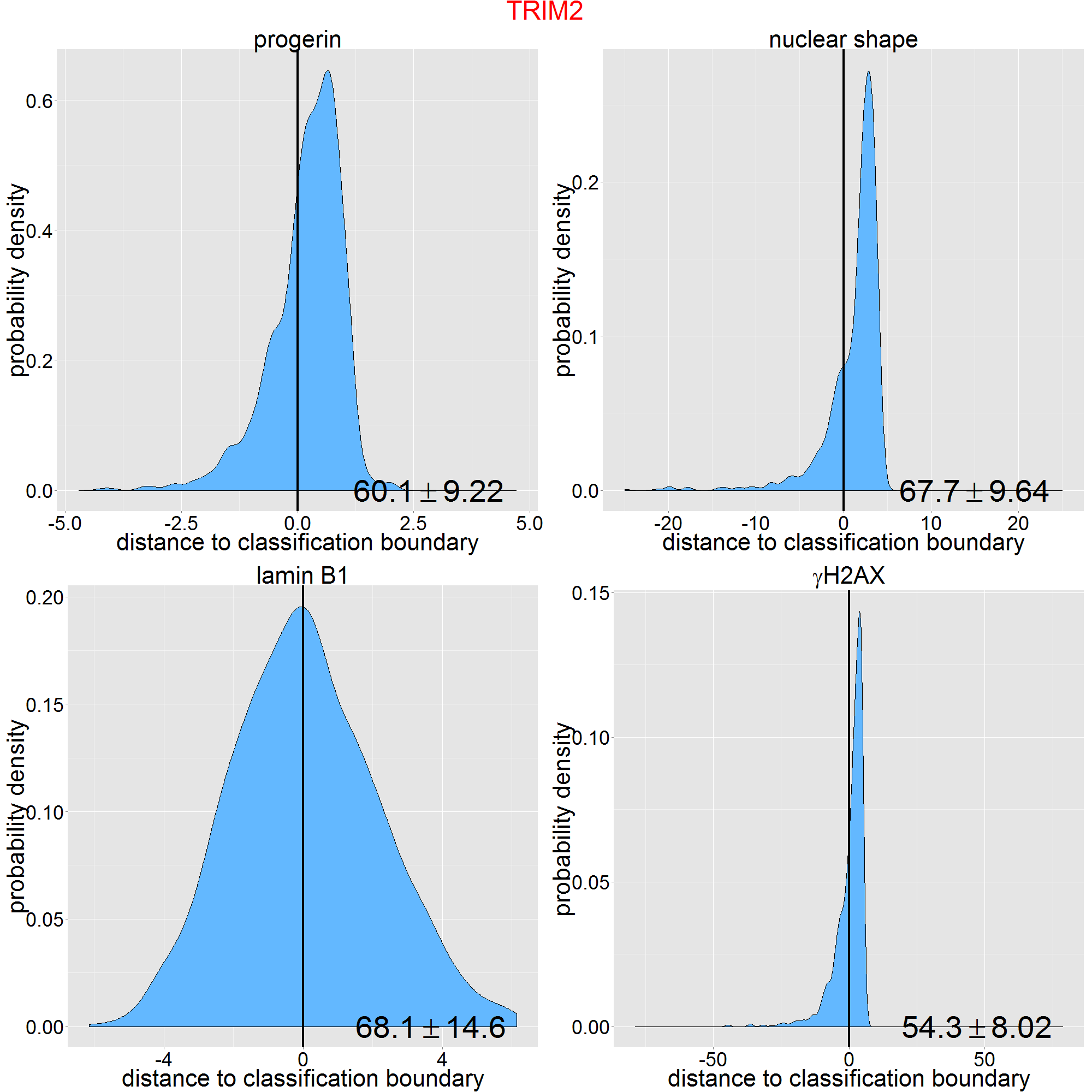


**Figure S6. An example of healthy-like cell percentage calculation for one siRNA: TRIM2.** Plotted here are the probability density distributions of all cells in TRIM2 sample in plate 1. Vertical lines are classification boundary, and numbers shown the average percentage of cells have positive (right, healthy-like) distance to the classification boundary with the standard deviations calculated from the 4 replicate plates. This average was later used for siRNA hits identification.

## S7 Table of selected siRNA hits

**siRNA hits in each channel.** Numbers in parenthesis show the percentage of healthy-like cells averaged over 4 independent replicate plates per siRNA well. Percentage of healthy-like cells of each channel in progeria controls are listed under channel name.

| Channel | siRNA hits |
| --- | --- |
| Nuclear Shape  (Progeria control baseline: 49%) | ASB12 (80%) UBE2D2 (79%) SKP2 (76%) LOC554251 (76%) RNF150 (75%) PCGF1 (74%) FLJ25076 (73%) FBXL10 (71%) |
| Lamin B1  (Progeria control baseline: 14%) | UBE2T (84%) CDC34 (82%) KUA-UEV (81%) UBE2O (81%) UBE2L6 (73%) PHF21B (73%) RNF39 (71%) FBXO8 (71%) HERC4 (70%) CUL5 (69%) RNF122 (69%) TRIM2 (68%) FBXO28 (68%) SMURF1 (68%) UBE3B (68%) FBXL11 (68%) RNF44 (68%) HERC3 (67%) HECTD1 (67%) UBE1L (66%) HERC5 (66%) PHF20 (66%) PHF11 (65%) FLJ25076 (65%) PHF17 (65%) FBXO38 (64%) NDP52 (64%) UBE2U (64%) WWP1 (64%) HERC2 (64%) ITCH (63%) RNF180 (62%) UBE2N (61%) TRIM55 (59%) ZMYND11 (58%) CUL7 (58%) DCUN1D4 (58%) VPS41 (58%) PRICKLE1 (57%) UBE2M (57%) TRIM52 (56%) SOCS2 (56%) UBE2D3 (56%) UBE2E2 (56%) RNF12 (56%) PDZRN3 (54%) LOC554251 (54%) LMO6 (53%) ARIH1 (53%) UBE2Q2 (52%) HERC6 (51%) UBE3A (51%) DCUN1D1 (50%) UBE2E3 (50%) WWP2 (50%) UBE2V2 (50%) UBE3C (50%) CBL (49%) TRIP12 (48%) RNF8 (48%) HECTD3 (46%) CUL4B (45%) INTS12 (45%) NEDD4L (45%) CUL2 (45%) DTX4 (44%) 39876 (43%) UBE2D1 (43%) RFPL2 (43%) ZNRF2 (42%) BMI1 (41%) FBXW8 (41%) MGRN1 (41%) HACE1 (41%) UBE2D4 (39%) |
| Progerin  (Progeria control baseline: 6%) | TIP120A (79%) WSB1 (70%) UBE2G2 (64%) WWP2 (62%) TRIM2 (59%) LOC554251 (55%) FBXO38 (52%) RNF39 (50%) TRIM55 (49%) MLLT6 (45%) FBXO17 (44%) FLJ25076 (44%) HERC3 (43%) CUL3 (43%) UBE2D2 (40%) FBXL13 (39%) TRIP12 (38%) UBE1C (37%) ASB5 (36%) SMURF1 (33%) RNF150 (33%) RNF44 (32%) FBXL11 (31%) PHF20L1 (30%) RNF32 (28%) ASB12 (27%) TRIM8 (26%) UBE2T (25%) HIP2 (23%) CBLC (23%) MYCBP2 (20%) |
| γH2AX (Progeria control baseline: 60%) | WWP2 (86%) WSB1 (81%) ZNF330 (78%) RFPL4B (77%) WDR24 (75%) |

## S8 Comparison with another method

We compared the results of our method with another multi-dimensional analysis method proposed in Ref [13]. We chose two channels: progerin and Lamin B1 for this comparison. To analyze our data using method in [13], we first randomly selected 5,000 cells (about 25%) from both GFP-progerin expressing and repressed controls respectively. We pooled these 10,000 cells together, and clustered them with GMM as described in [13]. In total, we identified 9 (8) clusters in progerin and γH2AX (lamin B1) channel, these were used as reference models for siRNA perturbation samples. We then computed probability of each cell belong to one of these 9 (8) clusters. Expectation of the proportion of cells inside each cluster for each perturbation is then calculated based on these probabilities. Using expected fraction of cells in each cluster as a vector, distance between each perturbation to GFP-progerin repressed (healthy-like) controls are calculated using KL divergence. Inverse of this divergence is then used as the metric to select important perturbations, the larger the metric the more similar to the healthy-like controls, hence the more important the perturbation. We then compared our metric (percentage of healthy-like cells) with this metric, and we found that they correlate well with each other.


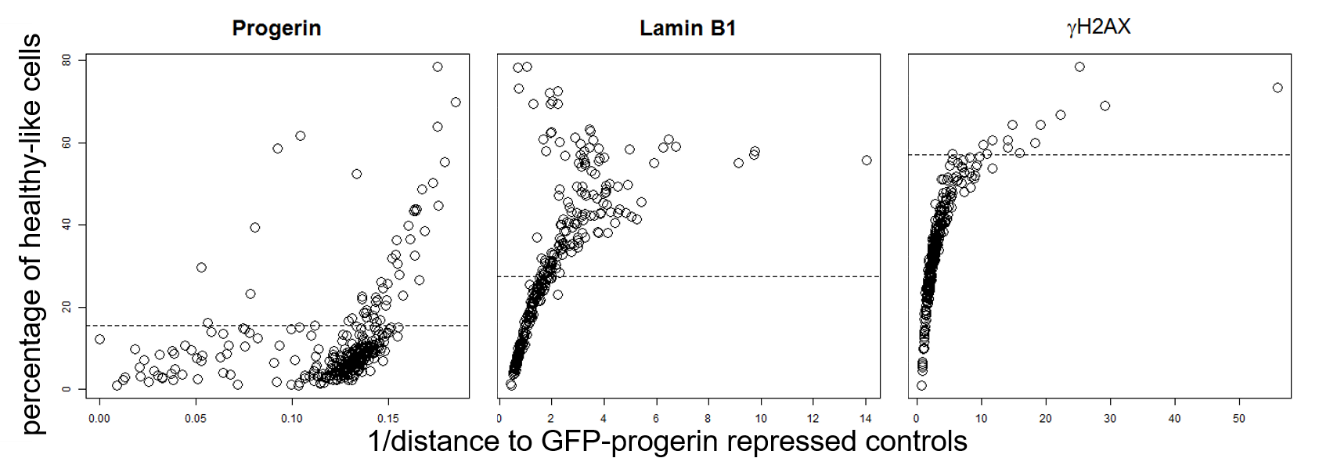


Figure S7. Comparison between our metric (healthy-like cell percentage, y axis) and metric derived using method proposed in [13] (x axis) in lamin B1, progerin and γH2AX channel. The two metrics correlates well in all channels, with Spearman correlation coefficient 0.98 for γH2AX channel, 0.91 for lamin B1 channel, 0.58 for progerin channel (p value << 0.05 in all cases).

## S9 List of screened siRNAs

## UBE2C SMURF1 HERC3 UBE2W DCUN1D3 BIRC6 CUL3 ITCH DCUN1D2 CUL1 UBE2O UBE2V1 UBE1C UBE2E3 UBE2U KUA-UEV EDD1 HIP2 UBE2V2 DCUN1D5 UBE2J2 HECW1 HUWE1 CAND2 UBE3B UBE2A UBE2R2 AKTIP UBE2D1 UBE3A TIP120A KIAA0317

## HECTD1 UBE2T HERC2 UBE2N UBE1L UBE1 UBE2D3 NEDD4 UBE2E2 HECTD3 UBE2E1 UBE3C TRIP12 HECTD2 UBE2D4 DCUN1D4 CDC34 FLJ34154 UBE2Z UBE2L3 HERC1 HACE1 UBE1DC1 UBE2M UBE2I UBE2Q2 TSG101 UBE2G2 CUL4A HERC4 CUL4B UBE2L6 DCUN1D1 CUL2 HERC5 UBE2NL CUL7 UBE2S UEVLD UBE2F HERC6 WWP1 SMURF2 NEDD4L HECW2 UBE2B UBE2J1 FLJ25076 UBE2Q1 UBE1L2 UBE2G1 FBXL15 UBE2H FBXO18 CUL5 LOC554251 ARIH1 FBXL19 UBE2D2 WSB1 WWP2 FBXL7 C10ORF46 ASB18

## FBXL16 FBXO27 FBXW10 FBXO42 FBXO21 FBXO40 FBXO30 FBXO17 FBXL10 SKP2 SOCS7 ASB12 FBXL11 WSB2 FBXL20 SOCS2

## FBXO43 LOC200933 ASB13 SPSB1 SOCS6 FBXO6 FBXL8 ASB16 LRRC29 FBXW8 ASB9 FBXO4 FBXO16 ASB14 FBXW11 ASB6

## RAB40C FBXW2 SPSB3 FBXO31 FBXO9 FBXL3P SPSB2 WDR71 FBXO41 SOCS5 FBXL13 ASB11 FBXO5 FBXL17 FBXO28 FBXO46

## LOC342897 FBXL18 SOCS3 FBXO15 FBXO3 FBXO22 FBXO11 FBXL14 FBXO7 FBXO25 RAB40A FBXL2 ASB5 CISH ASB8 FBXW9

## LOC652759 TULP4 NLRC5 FBXO8 CCNF FBXO44 LL0XNC01-237H1.1 ASB2 FBXO24 LOC440456 FBXO2 FBXO38 BTRC ASB7 FBXL12 FBXL3A

## ASB4 FBXO33 LGR6 FBXL6 NEURL2 ASB15 FBXO36 FBXW5 SOCS1 FLJ10916 FBXW12 FBXL4 FBXO32 SPSB4 FBXW7 FBXO39

## SHFM3 ASB10 FBXL5 ASB3 FBXO10 FBXL22 FBXO47 RAB40B FBXO34 MDM2 SOCS4 PRPF19 ASB17 TRIM6-TRIM34 ASB1 ZMYND11

## JARID1B RNF123 RKHD1 TRIM67 TRIM75 PHF17 OIT3 MGRN1 PHF7 TRIM39 RBX1 LOC653111 LOC642678 3/8/2009 BIRC3 MIB2

## PHF11 TRIM60 LOC644006 PHF6 TRIM42 BAHD1 RNF7 WDSUB1 TRIM41 RNF133 HRC MYCBP2 PHF20L1 RNF152 TRIM62 RNF125

## TRIM8 RNF122 TRIM63 RFPL4B WDR24 DTX4 PCGF1 TRIM3 KIAA1718 RNF32 PRICKLE1 CBL RFWD2 RSPRY1 BMI1 PHF20

## RNF39 RNF12 PDZRN3 C6ORF49 TRIM26 PHF21B ZNF645 RNF5 INTS12 ZNF592 CHD5 RNF180 UNKL MID2 ZNF313 RNF185

## RNF135 ZNRF2 PHF5A C20ORF18 3/4/2009 ANKIB1 PHF15 BRCA1 LOC92312 ZFAND6 PHF21A HR MLLT6 TRIM14 ZNRF3 NDP52

## LOC643904 TRIM40 LOC399937 TRIM43 LMO6 TRIM52 RNF144 LONRF1 SH3RF2 RNF150 PHF23 RNF25 RUFY1 ZNF330 UBR2 TRIM2

RFPL2 PHF16 ZNF179 RAD18 CBLC RNF44 TRIM55 BRPF3 PCGF3 RNF8 PHF13 DTX3L RNF148 VPS41 RNF103 TRIML1

## S10 Enlarged Figure 3


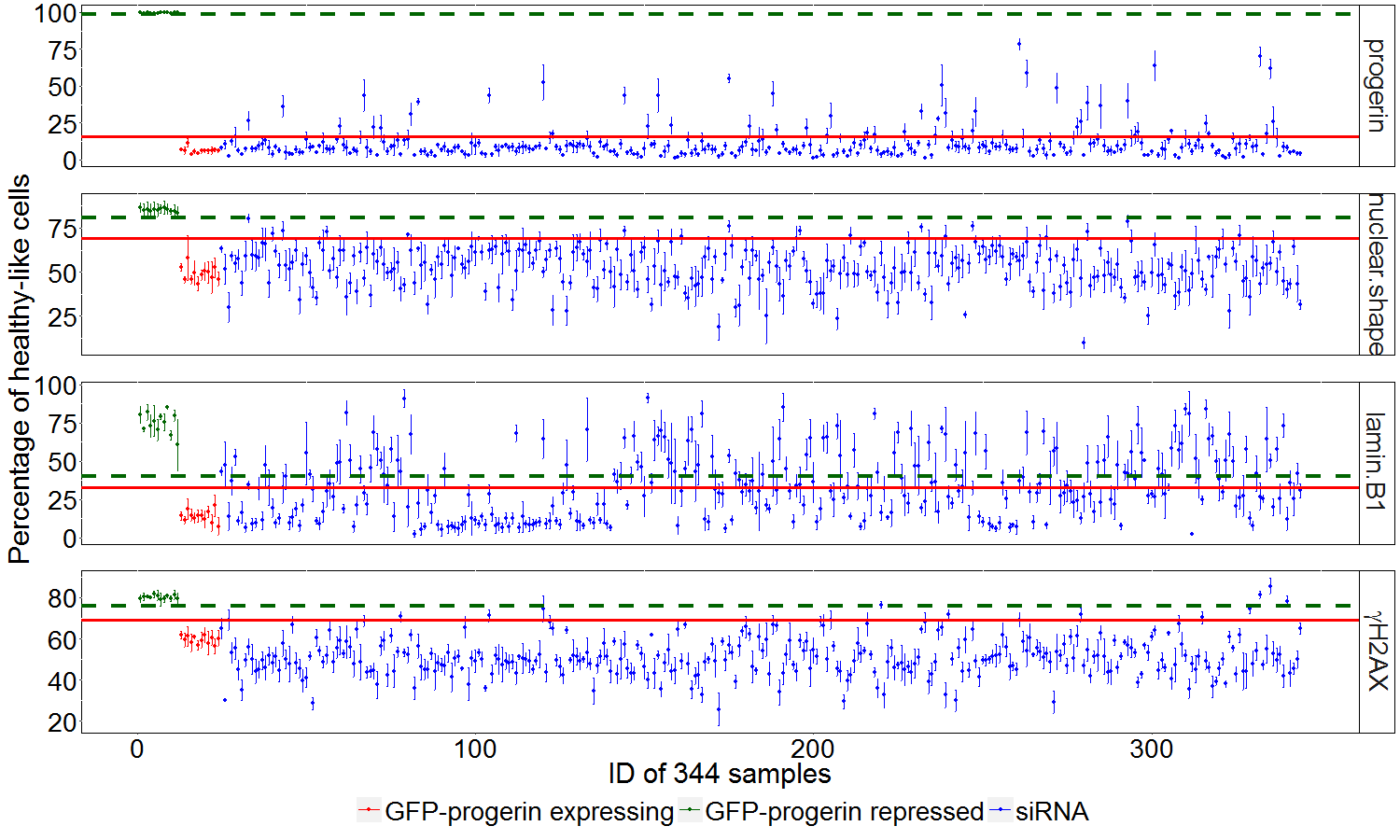


## S11 Enlarged Figure 5


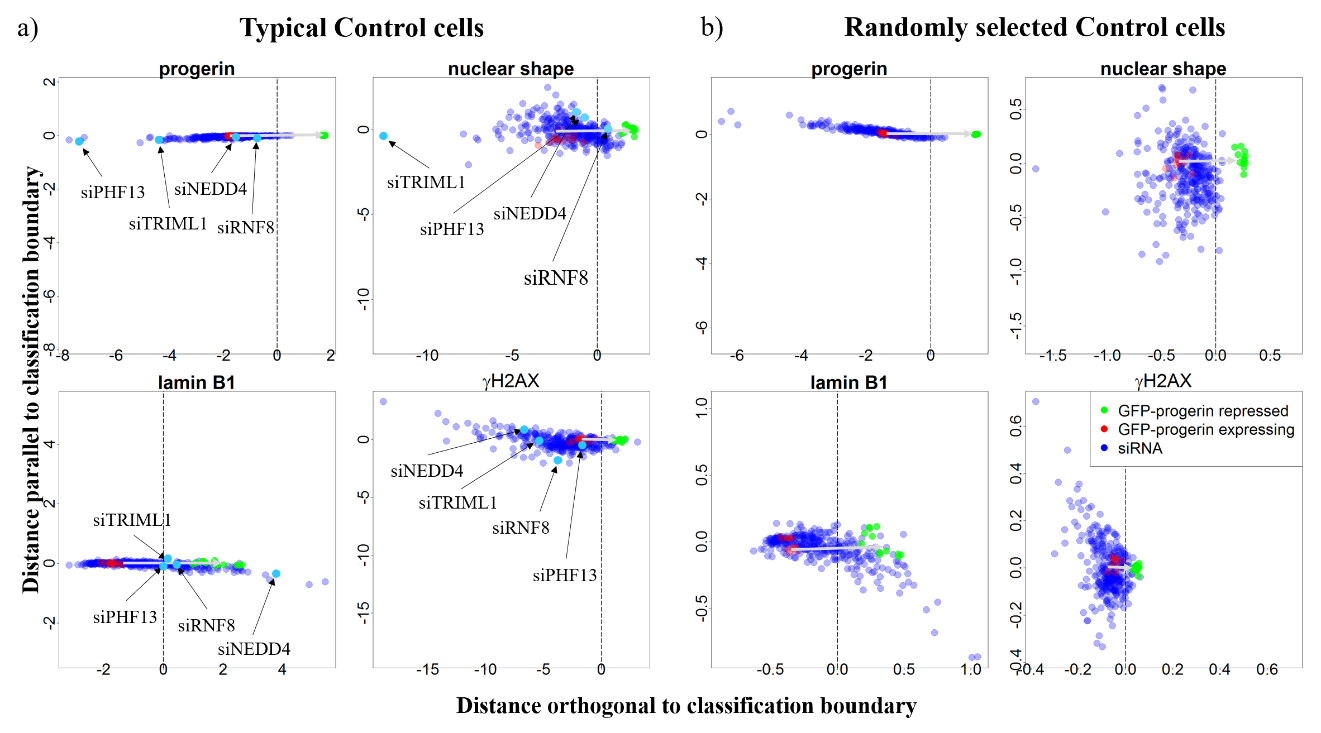

Supplement: Supplementary file 1 — Supplementary information. (DOCX 1694 kb) [file 12859_2018_2454_MOESM1_ESM.docx]
